# No-tillage and fertilization management on crop yields and nitrate leaching in North China Plain

**DOI:** 10.1002/ece3.1420

**Published:** 2015-02-17

**Authors:** Manxiang Huang, Tao Liang, Lingqing Wang, Chenghu Zhou

**Affiliations:** Key Laboratory of Land Surface Pattern and Simulation, Institute of Geographical Sciences and Natural Resources Research, Chinese Academy of SciencesBeijing, 100101, China

**Keywords:** Conventional tillage, crop yield, manure, no-tillage, straw, yield-scaled nitrate–nitrogen leaching loss

## Abstract

A field experiment was performed from 2003 to 2008 to evaluate the effects of tillage system and nitrogen management regimes on crop yields and nitrate leaching from the fluvo-aquic soil with a winter wheat (*Triticum aestivum L.)–*maize (*Zea mays L.)* double-cropping system. The tillage systems consisted of conventional tillage (CT) and no-tillage (NT). Three nitrogen management regimes were included: 270 kg N ha^−1^ of urea for wheat and 225 kg N ha^−1^ of urea for maize (U), 180 kg N ha^−1^ of urea and 90 kg N ha^−1^ of straw for wheat and 180 kg N of urea and 45 kg N ha^−1^ of straw for maize (S), 180 kg N ha^−1^ of urea and 90 kg N ha^−1^ of manure for wheat and 180 kg N ha^−1^ of urea and 45 kg N ha^−1^ of manure for maize (M). An array of tension-free pan lysimeters (50 cm × 75 cm) were installed (1.2 m deep) to measure water flow and 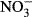-N movement. No significant effect of the N management regime on yields of winter wheat and maize grain was found in the 5-year rotation. Tillage systems had significant influences on 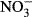-N leaching from the second year and thereafter interacted with N management regimes on 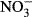-N loads during all maize seasons. The average yield-scaled 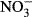-N leaching losses were in order of CTS < NTS< CTU < NTU <CTM < NTM, ranging from 0.88 (CTS) to 6.07 (NTM) kg N Mg^−1^ for winter wheat system and from 0.99 (CTS) to 6.27 (NTM) kg N Mg^−1^ for summer maize system for 5 rotation years. The results showed that CTS decreased the yield-scaled 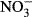-N leaching losses while sustaining crop grain yields. Considering the lower costs, NTS could be a potential alternative to decrease yield-scaled 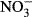-N leaching losses and improve soil fertility while maintaining crop yield for the winter wheat–maize double-cropping systems in the North China Plain.

## Introduction

Numerous studies have demonstrated that no-tillage is useful to decrease agriculture production costs, improve soil structure, increase organic carbon sequestration, reduce soil erosion (Dabney et al. 2004; Holland 2004), and maintain or increase crop yields (Ehlers and Claupein 1994; Baumhardt and Jones 2002). In contrast to these reports, no-tillage was less successful under conditions of high weed infestation (Soane and Ball 1998) or in heavy clay soils with little or no N fertilization (Rasmussen and Douglas 1992).

The effect of no-tillage on nitrate–nitrogen leaching loss is still a matter of controversy. Generally, no-tillage with surface mulch (crop straw or manure cover) may contribute to an increasing infiltration rate of soils, which may result in an increase in 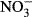-N leaching loss (Boddy and Baker 1990; Singh and Malhi 2006). On the other hand, some studies have found higher 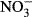-N leaching rates under CT due to increased N mineralization (Angle et al. 1993; Randall and Iragavarapu, 1995). The reported results of the effects of manure application on nitrate leaching are also inconsistent. Some studies have reported a higher potential risk of nitrate leaching for applications of manure in soils with high levels of soil organic matter (Chambers et al. 2000; Yan et al. 2002; Basso and Ritchie 2005). Zhou et al. (2014) demonstrated the beneficial reduction of nitrate leaching by 36% under synthetic N fertilizer (60% of applied N) plus pig manure (40% of applied N) compared with synthetic N fertilizer treatment in wheat–maize rotation system.

The North China Plain (NCP) is an important agronomic and animal husbandry area in China. At present, with an average annual total yield of the crops of winter wheat and summer maize reaching 15t ha^−1^, the NCP provides nearly 20% of the nation's grains. Since the 1990s, overfertilization has been common with the annual application rate of synthetic N in the NCP ranging from 450 to 800 kg N ha^−1^ year^−1^ for the typical winter wheat–summer maize double-cropping systems (Zhang et al. 2007). The intensive conventional tillage with excessive inputs of synthetic nitrogen fertilizer in this area has resulted in the decline of soil fertility and severe environmental pollution, especially 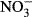-N leaching losses (Gao et al. 1999; Ju et al. 2006). Meanwhile, organic fertilizers (crop straw and manure) are the second N fertilizer resource in the NCP. Farmers have to deal with large quantities of crop straw produced from the double cropping and manure with a substantial amount of straw used for bedding that will have elevated nutrient levels from confined animal excreta (Yue 2009). It has been reported that the average manure N load is 183 kg N ha^−1^ in the agricultural areas of the NCP (Wu 2005). In general, returning crop straw to the field and manure application can increase soil C sequestration and N input (Baker et al. 2006). Nonetheless, large amounts of manure or crop straw retained on the soil surface can reduce crop yields (Kelley and Sweeney 2005). Especially, straw with a high C: N ratio decreases initial availability of nitrogen due to immobilization (Christensen 1985). Furthermore, there is a higher potential risk of nitrate leaching for excess application of manure (Andraski et al. 2000). Thus, it is necessary to include the N in straw and manure as sources of nutrients to reduce the use of mineral N fertilizer. However, little work has been done on these aspects for this area.

An appropriate nitrogen management regime in combination with a proper tillage system is expected to sustain soil fertility and agronomic productivity while decreasing yield-scaled 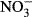-N leaching (Zhou and Butterbach-Bahl, 2014). It is known that the best management practices (BMPs) for fertilizers can decrease 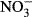-N leaching losses from fertilized fields while sustaining crop yield. The recommended chemical N rate with appropriate organic fertilizer (straw or manure) rate applied to wheat–maize double-cropping system under no-tillage may be an alternative of the conventional tillage fertilization systems in this region (Hu et al. 2006). A long-term field experiment was initiated in 2003 to investigate the effects of N fertilizer regimes and tillage systems on crop yields and yield-scaled 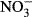-N leaching in the NCP.

## Materials and Methods

### Site description

A field experiment was conducted at Beiqiu (36°56′N, 116°36′E; 30 m above sea level) from October 2003 to September 2008, in Dezhou of Shandong province, China. The area has a semi-arid climate, with a long-term average annual precipitation of 585 mm (1961–2004). About 311-mm precipitation occurs during the rainy months of July–August. The average total precipitation of approximately 150 mm during the winter wheat growth season (mid-October to mid-June) is far less than the water demand of wheat. The soil was the fluvo-aquic soil (Shandong agricultural Department 1986), which was formed from the sediments of the Yellow River. The typical fluvo-aquic soil has a silt loam texture (sand, 13%; silt, 65%; clay, 22%) at 0 to 65 cm depth according to the USDA classification, a pH value of 8.1 to 8.4. The area is representative of the middle–high yield agricultural productivity in the NCP. The selected soil parameters were determined in October 2003 (Table1).

**Table 1:** Selected soil properties of experiment site.

Depth	BD^1^	OM^2^	Total N	P_2_O_5_	K_2_O	pH
cm	g cm^−3^	g kg^−1^				H_2_O
0–10	1.22	12.8	0.87	2.04	21.9	8.1
10–20	1.48	8.2	0.63	1.62	21.4	8.3
20–40	1.44	4.8	0.44	1.37	20.7	8.4
40–60	1.56	3.3	0.39	1.37	21.9	8.4

1 Soil bulk density.

2 Organic matter.

### Field experiment design

The experiment was a randomized, complete-block design with a split-plot arrangement of tillage systems as a main plot, three nitrogen regimes as subplots, and three replicates. The size of the main plot was 22.5 m × 40 m and that of subplots 6.5 m × 40 m. Summer maize in 2003 was planted without fertilization to make the soil fertility at the site homogeneous. There were two tillage systems: conventional tillage (CT) and no-tillage (NT). The CT consisted of fall plowing two times after maize harvest with a rototiller to a depth of about 0.2 m and harrowing two times to prepare the seedbed for wheat planting. There was no-tillage before maize seeding under CT, which is historically common in the Shandong province. The NT treatment used no soil disturbance except for planting. The practical nitrogen management regimes in local winter wheat–maize production were adopted in this study. The three nitrogen management regimes were as follows: 270 kg N ha^−1 ^year^−1^ (urea) for wheat and 225 kg N ha^−1 ^year^−1^ (urea) for maize (U), 180kg N ha^−1 ^year^−1^ (urea) and 90 kg N ha^−1^ year^−1^ (maize straw) for winter wheat and 180 kg N ha^−1^ year^−1^ (urea) and 45 kg N ha^−1^ year^−1^ (wheat straw) for maize (S), 180 kg N ha^−1^ year^−1^ (urea) and 90 kg N ha^−1^ year^−1^ (poultry manure) for wheat and 180 kg N ha^−1^ year^−1^ urea and 45 kg N ha^−1^ year^−1^ (poultry manure) for maize (M).

Urea for wheat was broadcast on the soil surface manually after rainfall or before irrigation. For U treatment, the urea (270 kg N ha^−1^) was in three equal split applications with one-third as basal fertilizer in late October (or early November) and the remaining as supplementary fertilizer in late March (or early April) and May in the following year. For S and M treatments, the amount of urea (180 kg N ha^−1^) was split into two equal for wheat in late October (or early November) and May in the next year. For CT treatments, urea applied as basal fertilizer for wheat was incorporated with tillage operation in the 20 cm. For maize, urea was manually broadcast on the soil surface after rainfall at early growing period.

On average, the poultry manure contained 22.5 g kg^−1^ total N, 4.09 g kg^−1^ alkali-hydrolysis N, 16.1 g kg^−1^ total P, 12.0 g kg^−1^ total K, 304 g kg^−1^ total C. The mean total C and N contents were 411.9 and 7.49 g kg^−1^ for winter wheat straw and 372.5 and 9.25 g kg^−1^ for maize straw, respectively. Details of poultry manure and crop straw composition are shown in Table2. Both wheat and maize straws were chopped into 5–8 cm and applied on the surface by hand. For S treatment, the maize straw was applied at 9.7 Mg ha^−1^ in late March for winter wheat and the wheat straw at 6.0 Mg ha^−1^ at the early growth period for maize. For M treatment, poultry manure was applied at 4 Mg ha^−1^ in late March for winter wheat and 2 Mg ha^−1^ at the early growth period for maize. For U plots, concentrated superphosphate and potassium sulfate were surface-broadcasted to plots at the rates of 120 kg P_2_O_5_ ha^−1^ and 170 kg K_2_O ha^−1^ for each crop, respectively. For S plots, 15.5 kg ha^−1^ (P) and 163.0 kg ha^−1^ (K) with maize straw were inputs for wheat, and 12 kg ha^−1^ (P) and 100 kg ha^−1^ (K) for maize with wheat straw. Therefore, 104.5 kg P ha^−1^ of concentrated superphosphate was applied to wheat, and 108 kg P ha^−1^ of concentrated superphosphate and 70 kg K ha^−1^ of potassium sulfate for maize. For M plots, 64 kg P ha^−1^ and 48 kg K ha^−1^ for winter wheat and 32 kg P ha^−1^ and 24 kg K ha^−1^ for maize with poultry manure applied were inputs; therefore, 56 kg P ha^−1^ of concentrated superphosphate and 122 kg K ha^−1^ of potassium sulfate for winter wheat and 88 kg P ha^−1^ of concentrated superphosphate and 146 kg K ha^−1^ of potassium sulfate for maize were applied, respectively.

**Table 2:** Composition of poultry manure and crop straw.

	Poultry manure	Winter wheat straw	Maize straw
	g kg^−1^		
C	304 (29.1)^1^	411.9 (46.8)	372.5 (38.9)
N	22.5 (3.8)	7.49 (1.6)	9.25 (2.7)
P	16.1 (1.9)	2.0 (0.3)	1.62 (0.5)
K	12.0 (2.8)	16.7 (1.9)	16.8 (4.9)

1 Numbers in parentheses are the standard errors.

**Figure 1:**
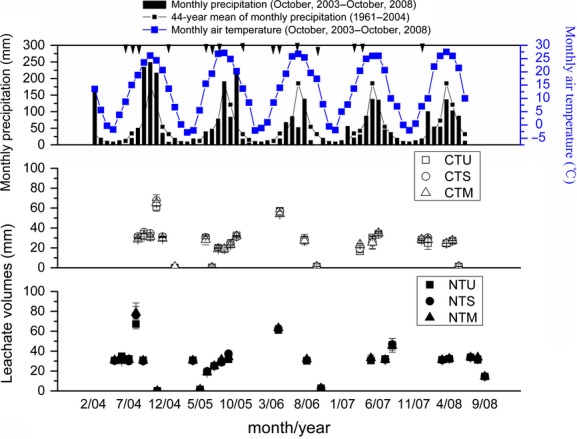
Monthly total precipitation (mm), monthly air mean temperature (°C), and monthly total leachate volumes (mm) collected from October 2003 to October 2008. Error bars are the standard errors of the treatment means. Solid arrows denote irrigation. CTU, urea (270 kg N ha^−1^) for wheat and urea (225 kg N ha^−1^) for maize with conventional tillage (CT); CTS, urea (180 kg N ha^−1^) and maize straw (90 kg N ha^−1^) for wheat and urea (180 kg N ha^−1^) and wheat straw (45 kg N ha^−1^) for maize with CT; CTM, urea (180 kg N ha^−1^) and poultry manure (90 kg N ha^−1^) for wheat and urea (180 kg N ha^−1^) and poultry manure (45 kg N ha^−1^) for maize with CT; NTU, urea (270 kg N ha^−1^) for wheat and urea (225 kg N ha^−1^) for maize with no-tillage (NT); NTS, urea (180 kg N ha^−1^) and maize straw (90 kg N ha^−1^) for wheat and urea (180 kg N ha^−1^) and wheat straw (45 kg N ha^−1^) for maize with NT; NTM, urea (180 kg N ha^−1^) and poultry manure (90 kg N ha^−1^) for wheat and urea (180 kg N ha^−1^) and manure (45 kg N ha^−1^) for maize with NT.

Seeding for winter wheat and maize was conducted manually under CT and NT systems. Winter wheat was seeded in rows at an interval of 20 cm at a rate of 180 kg ha^−1^ in mid-October except in 2003. Seeding of wheat was delayed until the 3rd of November by an abnormal precipitation of 166.9 mm in mid-October 2003. Maize was planted in mid-June at the density of 66,400 plants ha^−1^ in rows at an interval 60 cm. Irrigation water was applied using the surface flooding method with a water meter installed to control the irrigation rate on each plot. Winter wheat was irrigated with 75 mm water on each occasion on March 30, April 29, May 21 and October 15, 2004, and April 6, May 9, and Oct 30, 2005, and March 28, April 15 and October 14, 2006, and April 2 and May 5, 2007, and March 21, 2008. Summer maize was irrigated with 60 mm water on each occasion only on June 20, 2005 and July 30, 2006 (Fig.1). Weeds and insects were chemically controlled, that is herbicide (2, 4-Dichlorophenoxyacetic acid butylate) spraying within 1 week after sowing and insecticide (40% dimethoate, o, o–dimethyl S–[2-(methylamino)–2–oxoethyl] dithiophosphate) in May for winter wheat and late July for maize. Winter wheat was harvested (with DZ Combine harvester) during mid-June and maize in mid-October, with aboveground biomass removed for all treatments. The straws for S plots were conserved for use. The plant samples, comprising separate grain and straw from 2 m^2^ (1 m × 2 m) sampling area of wheat and 10 plants in the case of maize, were collected from the middle rows of each plot at harvest and were oven-dried at 65°C for 3 days. Grain and straw yields were recorded, and grain data were adjusted to 14% moisture content. The nitrogen content in straw of the subsamples of both wheat and maize and the poultry manure were determined using the micro-kjeldahl method by digesting the samples in H_2_SO_4_-H_2_O_2_ solution (Bremner and Mulvaney 1982).

Each plot was equipped with a large (0.50 m x 0.75 m) tension-free pan lysimeter in late-August, 2003 for subsurface flow measurements. A lysimeter was installed by excavating laterally from a trench border of each plot at the depth of 1.2 m. Winter wheat and maize roots mainly distribute in the top 0–1.0 m soil layer in the area (Liu et al. 2008; Qi et al. 2012). Therefore, the 1.2-m-deep lysimeter was enough for both winter wheat and maize crops. Lysimeter pan emplacement was made by excavating laterally from a trench a border (none yield row) of each plot at the 1.2 m depth. A lysimeter was inserted into the excavated area so that the pan edge was at least 0.15 m from the trench face. Each lysimeter was filled with coarse sand overlaid by medium-fine sand. The pans were pressed tightly by adjusting the turnbuckles beneath each lysimeter into the roof of the excavated area to ensure contact between the undisturbed soil above and the sand in the lysimeter to establish continuity with the soil profile (Zhu et al. 2002). This prevents wicking of water along the roof and around the lysimeter which was common with open face (empty) tension-free pan lysimeters. The design and installation of the pan lysimeters were similar to those described by Jemison and Fox (1992). Tubes were connected to transport water into buried 20-L storage bottles. All water in each bottle was siphoned out for analysis of nutrient concentrations, and the volume of flow was recorded. Generally, sampling was conducted manually once a week. When amounts of percolate were small, samples from two to four successive sampling times were pooled within a month before 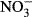-N analyses. After rainfall and irrigation events, samples of percolate were collected the next day when discharge occurred. The subsamples were stored frozen until analysis. Nitrate concentration was determined using a cadmium reduction–diazotization method on a flow-injection analyzer. The 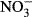-N leaching flux of each single percolation episode was calculated according to Zhou et al. (2014). The amount of seasonal leached 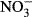-N was calculated as the sum of 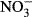-N in all samplings within the season. Flow-weighted average concentration values were weighted averages based on total NO_3_-N transport and total percolation for the corresponding period (Owens et al. 2000). The yield-scale 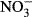-N leaching loss was defined as the seasonal 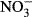-N leaching losses from a given treatment divided by the crop yield (kg N Mg^−1^ grain). Weather data were recorded at the meteorological station located 300 m away from the experimental site.

### Data analysis

Analysis of variance (ANOVA) for split-plot design was performed to determine the significance of differences among the treatments and interactions (Steel and Torrie 1986). In addition, differences for seasonal leachate volumes in millimeters of water depth (mm) among years were also evaluated. Then, multiple comparisons of overall treatment effects and interactions were made with a Fisher's protected least significant difference (LSD) test (*p *<* *0.05). Data on seasonal leachate volumes, 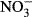-N leaching amounts, crop yields, and cumulate leachate volumes, 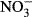-N concentration load, and yield-scaled 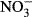-N leaching losses over 5 year met ANOVA assumptions and were analyzed without transformation.

## Results and discussion

### Crop yield

The results for crop grain yield for all the treatments are shown in Tables3 and 4. No significant effect on maize grain yield was observed due to tillage or N management regimes for the 5 years. The NT treatments appeared to have higher yields than CT treatments; however, the differences were not significant. Linden et al. (2000) found similar results in a 13-year study conducted on well-drained soils in east-central Minnesota. In that study, NT usually results in equal or greater corn yields than tillage treatments in the first 5 years of the study. However, after year 5, tillage treatments resulted in significantly greater yields than NT treatments. Our result was different from the decreased yield effect of conservation tillage on maize yield due to lower soil organic matter and poorly drained soils in Indiana, USA observed by Griffith et al. (1988). The effect of NT on crop yields varies depending on climate and soil type.

**Table 3:** Effect of tillage and nitrogen management regimes on summer maize grain yield (kg ha^−1^).

	N regimes			
Treatment	U	S	M	Mean
*2004*				
CT^1^	7257	7588	6938	7261
NT	6859	7549	7523	7310
Mean	7058	7569	7230	
LSD_0.05_: tillage = NS; N regime = NS; tillage × N = NS.				
*2005*				
CT	8298	8439	8551	8429
NT	8312	8586	8350	8416
Mean	8305	8513	8450	
LSD_0.05_: tillage = NS; N regime = NS; tillage × N = NS.				
*2006*				
CT	6527	6223	6644	6464
NT	6660	6579	7047	6762
Mean	6594	6401	6845	
LSD_0.05_: tillage = NS; N regime = NS; tillage × N = NS.				
*2007*				
CT	6265	5755	5914	5978
NT	6445	5993	5794	6077
Mean	6355	5874	5854	
LSD_0.05_: tillage = NS; N regime = NS; tillage × N = NS.				
*2008*				
CT	7688	8625	7762	8025
NT	8027	8604	7801	8144
Mean	7858	8614	7781	
LSD_0.05_: tillage = NS; N regime = NS; tillage × N = NS.				
*2004–2008*				
CT	36,035	36,629	35,810	54,237
NT	36,304	37,312	36,513	55,064
Mean	36,170	36,970	36,161	
LSD_0.05_: tillage = NS; N regime = NS; tillage × N = NS.				

1 CT, conventional tillage, the soil is tilled with a rototiller in the top soils of 20 cm before winter wheat sowing in autumn. There was no-tillage before maize seeding. NT, no-tillage. U, urea (270 kg N ha^−1^) for wheat and urea (225 kg N ha^−1^) for maize. S: urea (180 kg N ha^−1^) and maize straw (90 kg N ha^−1^) for wheat and urea (180 kg N ha^−1^) and wheat straw (45 kg N ha^−1^) for maize. M: urea (180 kg N ha^−1^) and poultry manure (90 kg N ha^−1^) for wheat and urea (180 kg N ha^−1^) and poultry manure (45 kg N ha^−1^) for maize.

**Table 4:** Effect of tillage and nitrogen management regimes on winter wheat grain yield (kg ha^−1^)

	N regimes			
Treatment	U	S	M	Mean
*2003–2004*				
CT^1^	4144	3801	3928	3958
NT	3943	3777	3744	3821
Mean	4043	3789	3836	
LSD_0.05_: tillage = NS; N regime = NS; tillage × N = NS.				
*2004–2005*				
CT	5865	5810	5632	5769
NT	5583	5478	5575	5546
Mean	5724	5644	5604	
LSD_0.05_: tillage = NS; N regime = NS; tillage × N = NS.				
*2005–2006*				
CT	5407	5060	5202	5223
NT	4745	4855	4750	4783
Mean	5076	4958	4976	
LSD_0.05_: tillage = 428; N regime = NS; tillage × N = NS.				
*2006–2007*				
CT	6580	6440	6512	6511
NT	5644	5131	5218	5331
Mean	6112	5785	5865	
LSD_0.05_: tillage = 709; N regime = NS; tillage × N = NS.				
*2007–2008*				
CT	6773	6582	6684	6680
NT	6445	6019	6205	6223
Mean	6609	6301	6444	
LSD_0.05_: tillage = NS; N regime = NS; tillage × N = NS.				
*2003–2008*				
CT	28,769	27,693	27,958	28,140
NT	26,360	25,260	25,492	25,704
Mean	27,565	26,476	26,725	
LSD_0.05_: tillage = 1862; N regime = NS; tillage × N = NS.				

1 The treatment details are the same as those in Table3.

During the first 2 years of the field experiments, there was insignificant difference in winter wheat yields among the different treatments. After this initial lag period, the tillage effect was observed in the third and fourth years. The wheat grain yield in the NT treatments was lower by 8.4% in the third year and by 18.1% in fourth year compared with CT treatments, respectively. Tillage in autumn (CT) generally benefits winter wheat roots development in deeper soil profile and absorbs subsoil water in serious dry season in the NCP (Dong et al. 2007). In the fifth year, the wheat grain yield in the NT treatments was also lower than that of the CT treatments, although the difference was insignificant. The higher than average precipitation in April 2007 may offset the shortage of NT on growth of winter wheat, which implies that the effect of NT on yield of winter wheat was affected by the rainfall pattern in dry growing season.

There was significant tillage effect on cumulative wheat yields (Table4). The NT treatments decreased cumulative wheat yield by 12.2% with annual average of 2.4%. Our results were consistent with other experiments conducted in the NCP, which have demonstrated that compared with traditional tillage farming, NT could reduce winter wheat grain yield (Li et al. 2008; Peng et al. 2011). The negative effects of NT on winter wheat grain yield could be related to the low germination rate and poor plant growth caused by NT (Wuest et al. 2000; Hemmat and Taki 2001). In contrast to our results, conservation tillage's positive effect on rainfed winter wheat yield was observed under the Chinese Loess Plateau climate due to improved soil water storage under monoculture conditions (Su et al. 2007; Wang et al. 2009).

No significant effect on seasonal maize and winter wheat grain yield or cumulative crop grain yields was observed due to N management regime throughout 5 years (Tables4). Although CTS decreased cumulative wheat yield by 3.7% with annual average of 0.7% compared with CTU, the difference was insignificant. These results suggest that there is greater potential to decrease the rates of urea application (33.3% reduction for wheat and 20% for maize) while sustaining crop grain yields by adopting S and M regimes in place of the U regime in the area studied.

### Percolation

The monthly total percolation was showed in Figure1. There were only two drainage episodes in the autumn of 5 winter wheat seasons, one in October 2004 and the other one in October 2006 after irrigation. Generally, no measurable percolation occurred from October to the February of the subsequent year due to dry and freezing weather conditions during the experiment period. Most of percolation occurred as a consequence of successive episodes of irrigation or heavy rainfall from March to September, especially during rainy summer, which agrees with the results reported in other studies (Huang et al. 2011; Zhou et al. 2012). The seasonal leachate volumes were different among years due to rainfall (Tables6). The total leachate volumes during maize seasons varied from the lowest amount in year 2006 to the highest amount in year 2004 (Table6), corresponding to total precipitation during maize seasons of a minimum of 242 mm in 2006 to a maximum of 654 mm in 2004 (Fig.1), respectively.

**Table 5:** Effect of tillage and nitrogen management regimes on leachate volumes (mm) during winter wheat seasons.

	N regimes			
Treatment	U	S	M	Mean
*2003–2004*				
CT^1^	30.6b^2^	31.2b	28.8b	30.2
NT	30.7c	30.4c	30.9b	30.7
Mean	30.7	30.8	29.9	
LSD_0.05_: tillage = NS; N regime = NS; tillage × N = NS.				
*2004–2005*				
CT	30.8b	30.9b	28.5b	30.1
NT	32.6b	31.7bc	32.4b	32.2
Mean	31.6	31.3	30.5	
LSD_0.05_: tillage = NS; N regime = NS; tillage × N = NS.				
*2005–2006*				
CT	57.1a	56.3a	54.1a	55.8
NT	61.2a	62.3a	63.2a	62.2
Mean	59.1	59.3	58.7	
LSD_0.05_: tillage = 2.8; N regime = NS; tillage × N = NS.				
*2006–2007*				
CT	17.7c	20.7c	24.5b	21
NT	33.2b	34.1b	32.9b	33.5
Mean	25.5	27.4	28.8	
LSD_0.05_: tillage = 3.2; N regime = NS; tillage × N = NS.				
*2007–2008*				
CT	53.1a	58.7a	56.6a	56.1
NT	63.8a	63.4a	63.9a	63.7
Mean	58.4	61.1	60.2	
LSD_0.05_: tillage = 5.2; N regime = NS; tillage × N = NS.				

1 The treatment details are the same as those in Table3.

2 For the same treatment within a column, different letters indicate significant difference between years at P < 0.05.

**Table 6:** Effect of tillage and nitrogen management regimes on leachate volumes (mm) during summer maize seasons

	N regimes			
Treatment	U	S	M	Mean
*2004*				
CT^1^	155.8a^2^	169.0a	157.8a	160.9
NT	165.4a	170.3a	170.9a	168.9
Mean	160.6	169.7	164.4	
LSD_0.05_: tillage = NS; N regime = NS; tillage × N = NS.				
*2005*				
CT	95.1b	94.3b	92.7b	94.1
NT	105.3b	110.9b	109.3b	108.5
Mean	100.2	102.6	100.9	
LSD_0.05_: tillage = 5.2; N regime = NS; tillage × N = NS.				
*2006*				
CT	28.4e	28.3d	27.2d	27.9
NT	30.1d	30.8d	31.9c	31
Mean	29.2	29.6	29.6	
LSD_0.05_: tillage = 2.5; N regime = NS; tillage × N = NS.				
*2007*				
CT	64.7c	59.1c	60.4c	61.3
NT	78c	78.3c	76.7d	77.6
Mean	71.4	68.6	68.5	
LSD_0.05_: tillage = 12.7; N regime = NS; tillage × N = NS.				
*2008*				
CT	53.1d	52.9c	54c	53.2
NT	80c	79.5c	78.9c	79.5
Mean	66.4	67	66.3	
LSD_0.05_: tillage = 6.0; N regime = NS; tillage × N = NS.				

1 The treatment details are the same as those in Table3.

2 For the same treatment in a column, different letters indicate significant difference between years at P < 0.05.

The effect of N management regime on leachate volume was insignificant throughout the 5-year rotation (Tables7). The tillage effects on leachate volumes were insignificant for the first three cropping seasons (P > 0.05). Thereafter, tillage significantly affected seasonal leachate volumes (Tables6).

**Table 7:** Effect of tillage and N management regimes on 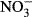-N leaching (kg 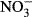-N ha^−1^) during winter wheat seasons

	N regimes			
Treatment	U	S	M	Mean
*2003–2004*				
CT^1^	3.2	1.7	8.4	4.4
NT	3.9	2.7	12.5	6.4
Mean	3.6	2.2	104	
LSD_0.05_: tillage = NS; N regime = 1.8; tillage × N = NS.				
*2004–2005*				
CT	3.1	1.3	13.3	5.9
NT	3.9	1.8	16.8	7.5
Mean	3.5	1.6	15.0	
LSD_0.05_: tillage = 0.6; N regime = 1.9; tillage × N = NS.				
*2005–2006*				
CT	25.3	12.8	47.2	28.4
NT	31.5	18.8	60.7	37.0
Mean	28.4	15.8	54.0	
LSD_0.05_: tillage = 10; N regime = 4.1; tillage × N = NS.				
*2006–2007*				
CT	2.2	0.5	8.6	3.8
NT	5.1	1.7	17.2	8.0
Mean	3.7	1.1	12.9	
LSD_0.05_: tillage = 4.1; N regime = 4.4; tillage × N = NS.				
*2007–2008*				
CT	13.7	8.1	34.7	18.8
NT	27.6	10.4	47.3	28.4
Mean	20.6	9.3	41.0	
LSD_0.05_: tillage = NS; N regime = 4.7; tillage × N = 6.7.				

1 The treatment details are the same as those in Table3.

There were about 100 mm of the 5-year cumulative leachate volumes for NT subplots more than CT subplots (Table7), which could be related to improved pathways for water percolation and the mechanical delay of runoff by rough surface to increase more water moving into the soil matrix during rain-runoff events in NT plots (Wilson et al. 2004). The temporal lag effect of NT system on water percolation observed in this study reflected the changes of soil hydraulic properties under NT treatment, which may be a very slow process. Higher biological activity, increased soil organic matter due to the retention of organic material as crop residues and manure on the soil surface, and reduced soil disturbance under NT plots lead to a more stable soil pore system (Six et al. 2002). An improved soil structure and continuous soil pores enable higher infiltration and ultimately increased available water for winter wheat production (Thierfelder and Wall 2009; Liu and Luo 2011).

### Nitrate–nitrogen concentration and leaching

**Figure 2:**
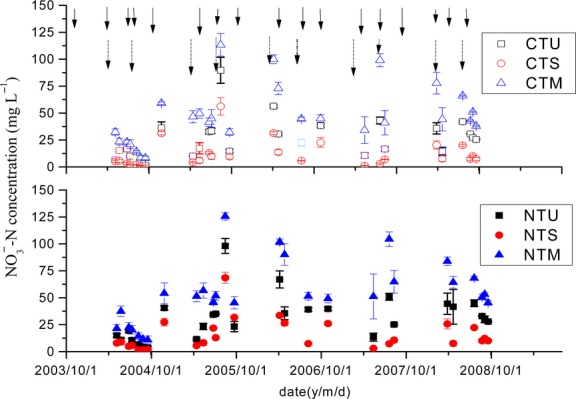
The NO_3_-N concentration (mg L^−1^) in leachate from lysimeters under different treatments by collection date, November 2003 to October 2008. Error bars are the standard errors of the treatment means. Solid arrows denote urea application, and short dash dot arrows denote straw or manure application. The treatment details are the same as those in Figure1.

Leaching 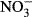-N concentrations displayed strong variation within sampling dates and significantly different between treatments (Fig.2). Generally, the 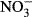-N concentrations in leachate had higher levels after fertilizer application in April and late July/early August followed irrigation or rainfall. The 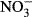-N concentrations in leachate in 2003–2004 rotational year were relative lower likely due to no fertilization of NPK during maize season in 2003 and dilution of abundant percolation during 2004 summer seasons. Manure application even significantly raised the 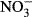-N concentration under the M plots from the first winter wheat season compared to the other treatments (P < 0.05). The highest concentration of 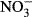-N in leachate was measured in the manure plot while the lowest values were always observed in the S plot for both tillage systems (Table7). The influences of application of both straw and poultry manure on the concentration of NO_3_-N in leachate were different. Studies found that there were more 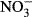 subject to leaching from manure N sources than those from inorganic N sources and inverse for S treatment (Bergström and Kirchmann 1999; Thomsen 2005). The NO_3_-N concentration values of the leachate were relative lower under the CT than those of the NT (Table7). This may be related to reduced crop uptake of 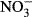 due to undeveloped crop roots in subsurface under NT conditions (Qin et al. 2004) and more 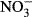 coming from mineralization of organic N accumulated in soil surface of NT plots (Thomsen and SØrensen 2005).

There was significant interaction between tillage and N management regimes on 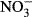-N concentration in leachate (Table7). Flow-weighted average 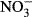-N concentration (FWC) across the 5 years for CTS was the lowest with 9.6 mg/L across the 5-year rotation, which was lower than the EPA critical level of 10 mg/L, followed by NTS. FWC of NTM was the highest with 52.8 mg/L, followed by CTM treatment.

Nitrate N leakage generally occurred after N fertilizer application following irrigation or/and rainfall during spring and summer in our experiment. There were only two episodes of nitrate N losses occurring from late autumn through overwinter months across 5 rotation years (Figs.2). Field observation in the NCP ever illustrated that no leaking occurred under the irrigation amount of 75 mm during winter wheat season assuming 1 m as the root zone (Yuan et al. 1995).

N management regimes had significant influences on 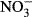-N leaching during all seasons (Tables9). The 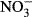-N leaching for S treatments was relatively low, while the highest was found for treatments of M. Compared with U plots, the total amount of 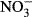-N leaching from S treatments over 5 years was lower by 55.9% and greater by 85% for M treatments (Table7). It is understood that the C:N ratios of organic materials applied in field are the major factors determining the immobilization and subsequent mineralization and release of N in response to organic material inputs (Whitmore 1996). Generally, the C:N ratios > 30 are conducive to N immobilization and < 20 for net N mineralization. While the N input rate in three N management regimes was equal in our study, C input and the C:N ratios were different. The yearly total C input in S treatment was 14.2 Mg ha^−1^ with C:N ratios of 55.0 for wheat straw and 40.3 for maize straw. For M treatment, the yearly total C input was 6 Mg ha^−1^ with the C: N ratio of 13.5. Christensen (1985) reported N immobilization of 1–3 kg per Mg straw in straw application field. Murphy et al. (2000) found that both soluble organic and mineral N pools were smaller in the soil amended with maize residues (C:N = 108:1) due to immobilization. Poudel et al. (2002) reported high rates of N mineralization and increased soil microbial activity in manure application soil. Therefore, it is most likely to immobilize N and lower the level of nitrate leaching in S plots while there was high rate of N mineralization and leaching in M plots.

**Table 8:** Effect of tillage and N management regimes on 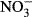-N leaching (kg 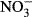-N ha^−1^) during maize seasons.

	N regimes			
Treatment	U	S	M	Mean
*2004*				
CT^1^	13.4	3.4	24	13.6
NT	15.1	5.3	26.5	15.6
Mean	14.2	4.4	25.3	
LSD_0.05_: tillage = NS; N regime = 2.3; tillage × N = NS.				
*2005*				
CT	39.0	20.9	52.9	37.6
NT	51.5	32.1	76.6	53.4
Mean	45.3	26.5	64.7	
LSD_0.05_: tillage = 3.7; N regime = 3.9; tillage × N = 5.5.				
*2006*				
CT	6.3	1.6	12.2	6.7
NT	11.8	2.3	16.5	10.2
Mean	9.0	2.0	14.3	
LSD_0.05_: tillage = 1.1; N regime = 1.7; tillage × N = 2.4.				
*2007*				
CT	18.8	3.2	39.6	20.5
NT	27.9	7.3	61.7	32.3
Mean	23.4	5.2	50.7	
LSD_0.05_: tillage = 8.6; N regime = 4.3; tillage × N = 6.1.				
*2008*				
CT	19	7	28.5	18.2
NT	29.8	12.4	46.3	29.5
Mean	24.4	9.7	37.4	
LSD_0.05_: tillage = 4.2; N regime = 1.2; tillage × N = 1.7.				

1 The treatment details are the same as those in Table3.

**Table 9:** Effect of tillage and nitrogen management regimes on cumulative leachate volumes (mm), 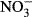-N leachate loss (kg 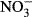-N ha^−1^) and flow-weighted average 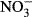-N concentration (mg L^−1^) for 5 year

	N regimes			
Treatment	U	S	M	Mean
*Leachate volumes (mm)*				
CT^1^	615	632	615	621
NT	711	724	722	719
Mean	663	678	669	
LSD_0.05_: tillage = 8; N regime = NS; tillage × N = NS.				
*Concentration (mg L*^*−1*^*)*				
CT	23.3	9.6	43.7	25.6
NT	29.3	13.0	52.8	31.7
Mean	26.3	11.3	48.3	
LSD_0.05_: tillage = 1.79; N regime = 1.09; tillage × N = 1.54.				
*NO*_*3*_*-N leaching (kg NO*_*3*_*-N ha*^*−1*^*)*				
CT	143.9	60.7	269.3	157.9
NT	208.2	94.8	381.9	228.3
Mean	176.1	77.7	325.7	
LSD_0.05_: tillage = 12.9; N regime = 9.5; tillage × N = 13.5.				

1 The treatment details are the same as those in Table3.

NT had significant influences on 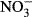-N leaching from the second year (Tables9). The total amount of 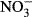-N leaching from NT system over 5 years was greater by 44.6% compared to that from CT system (Table7), which may be due to more percolate volumes and increased potential for transport of 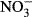 to groundwater under NT compared to CT (Bronick and Lal 2005).

The tillage system and N management regimes interaction on 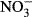 leaching was significant (Tables9). From the second year, tillage systems interacted with N management regimes on 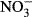-N loads for maize seasons (Table9). Interactive effect of tillage and N management regimes on 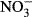-N leaching was also observed in 2007/2008 for wheat season. This was probably due to enhanced soil water percolation by adequate rainfall of 100.6 mm in April 2007 in comparison with the 44-year average of 34 mm in the same period (Fig.1). For the 5-year cumulative 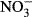-N leaching, the highest losses were measured in the treatments NTM and CTM, followed by NTU and CTU, and then by NTS and CTS (Table7). As mentioned above, there were higher macropores and biochannels and more 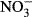 in the manure application soil under NT condition, which might cause more water and 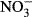 movement under NTM plots. Averagely, the leaching loss rates of N applied ranged from 2.5% (CTS) to 15.4% (NTM), which were lower than the average level of 19.1% derived from the values of 22% and 15% of applied N to wheat and maize systems worldwide (Zhou and Butterbach-Bahl 2014). In contrast to our results, some studies (Zavattaro et al. 2012; Zhou et al. 2014) reported that manure application and NT decreased 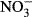-N leaching. They attributed the decreasing of 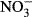-N leaching to increased immobilization of nitrogen under manure surface application and incorporation into soil and higher level of ammonia losses (30% of N applied) from surface applied urea under NT system. In our experiment, fertilizer including urea and manure or crop straw is surface applied, which decreased immobilization of nitrogen. Moreover, the ammonia loss rates are about 3–8% of N applied at the rates of 120–360 kg N ha^−1^ of urea (Wang et al. 2002) and 19.5% under poultry application at the rate of 24 Mg N ha^−1^ (Li et al. 2009). Therefore, surface application increased the amount of N sensitive to leaching loss from manure treatment in our experiment.

### Yield-scaled basis NO_3_^−^-N leaching losses

Nitrogen management regimes had significant influences on yield-scaled 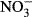-N leaching during all seasons (Tables11). The values for S treatments were lowest, while the highest was found for the M treatment. NT significantly increased yield-scaled 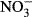-N leaching losses from the second year and had interaction with N management regimes from 2006 to 2008 maize season (Table11).

**Table 10:** Effect of tillage and N management regimes on yield-scaled 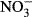-N leaching (kg 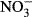-N Mg^−1^ grain) during winter wheat seasons.

	N regimes			
Treatment	U	S	M	Mean
*2003–2004*				
CT^1^	0.78	0.44	2.14	1.12
NT	1.00	0.71	3.35	1.69
Mean	0.89	0.58	2.74	
LSD_0.05_: tillage = NS; N regime = 0.54; tillage × N = NS.				
*2004–2005*				
CT	0.53	0.23	2.34	1.03
NT	0.71	0.33	3.05	1.36
Mean	0.62	0.28	2.69	
LSD_0.05_: tillage = 0.6; N regime = 0.36; tillage × N = NS.				
*2005–2006*				
CT	4.69	2.53	9.08	5.43
NT	6.64	3.87	12.80	7.77
Mean	5.66	3.20	10.94	
LSD_0.05_: tillage = 0.76; N regime = 1.01; tillage × N = NS.				
*2006–2007*				
CT	0.34	0.08	1.31	0.58
NT	0.92	0.33	3.21	1.49
Mean	0.63	0.20	2.26	
LSD_0.05_: tillage = 0.63; N regime = 0.59; tillage × N = 0.83.				
*2007–2008*				
CT	2.01	1.23	5.20	2.81
NT	4.31	1.73	7.61	4.55
Mean	3.16	1.48	6.41	
LSD_0.05_: tillage = 2.17; N regime = 0.73; tillage × N = 1.03.				
*2003–2008*				
CT	1.65	0.88	4.01	2.18
NT	2.74	1.40	6.07	3.40
Mean	2.19	1.14	5.04	
LSD_0.05_: tillage = 0.63; N regime = 0.30; tillage × N = 0.42.				

1 The treatment details are the same as those in Table3.

**Table 11:** Effect of tillage and N management regimes on yield-scaled 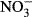-N leaching (kg 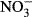-N Mg^−1^ grain) during maize seasons.

	N regimes			
Treatment	U	S	M	Mean
*2004*				
CT^1^	1.87	0.46	3.49	1.94
NT	2.25	0.71	3.53	2.16
Mean	2.06	0.58	3.51	
LSD_0.05_: tillage = NS; N regime = 0.43; tillage × N = NS.				
*2005*				
CT	4.81	2.52	6.19	4.51
NT	6.24	3.75	9.22	6.41
Mean	5.53	3.14	7.71	
LSD_0.05_: tillage = 0.60; N regime = 0.91; tillage × N = NS.				
*2006*				
CT	0.96	0.27	1.83	1.02
NT	1.78	0.35	2.33	1.49
Mean	1.37	0.31	2.08	
LSD_0.05_: tillage = 0.15; N regime = 0.16; tillage × N = 0.22.				
*2007*				
CT	3.04	0.55	6.67	3.41
NT	4.34	1.21	10.76	5.44
Mean	3.69	0.88	8.81	
LSD_0.05_: tillage = 0.63; N regime = 0.88; tillage × N = 1.24.				
*2008*				
CT	2.48	0.82	3.71	2.34
NT	3.72	1.44	5.96	3.71
Mean	3.10	1.13	4.83	
LSD_0.05_: tillage = 0.89; N regime = 0.40; tillage × N = 0.57.				
*2004–2008*				
CT	2.69	0.99	4.39	2.69
NT	3.75	1.59	6.23	3.86
Mean	3.22	1.29	5.31	
LSD_0.05_: tillage = 0.18; N regime = 0.18; tillage × N = 0.25.				

1 The treatment details are the same as those in Table3.

The yield-scaled 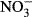-N leaching losses ranged from 0.08 to 12.8 kg N Mg^−1^ (mean: 2.78 kg N Mg^−1^) and 0.27 to 10.76 kg N Mg^−1^ (mean: 3.24 kg N Mg^−1^) for winter wheat and maize systems, respectively, which were within the scope of 0.3 to 15.1 kg N Mg^−1^ and 0.3 to 25.6 kg N Mg^−1^ for wheat and maize systems reported by Zhou et al. (2014). On average, the rather low values in CTS (0.08–2.53 kg N Mg^−1^, mean: 0.90 kg N Mg^−1^) and the rather higher values in NTM (3.05–12.80 kg N Mg^−1^, mean: 6.0 kg N Mg^−1^) were observed in winter wheat system. Similarly, the rather low levels in CTS (0.27–2.52 kg N Mg^−1^, mean: 0.92 kg N Mg^−1^) and the rather higher levels in NTM (2.33–10.76 kg N Mg^−1^, mean: 6.36 kg N Mg^−1^) in maize system were observed. Obviously, the yield-scaled 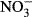-N leaching losses for CTS were lower than the value of 3.47 to 6.71 kg N Mg^−1^ for wheat system with N fertilization rate being in the ranges of > 250 kg N ha^−1^ and 2.82 to 6.18 kg N Mg^−1^ for maize system with N fertilization rate being in the ranges of 200 to 300 kg N ha^−1^ reported by Zhou et al. (2014).

The tillage and N management regimes had markedly influences on the average yield-scaled 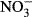-N leaching losses across 5 years (Tables11). Under winter wheat system, the average yield-scaled 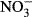-N leaching losses for CTS were significantly lower compared to CTU, while the value for NTS was lower than that of CTU, but the difference was not significant. Under maize system, CTS and NTS significantly decreased the average yield-scaled 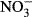-N leaching losses as compared to CTU. These showed that accepting yield losses of 0.7% for CTS and 2.4% for NTS compared to CTU would decrease the 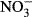-N leaching losses per kg wheat yield to a certain extent. In the maize systems, both CTS and NTS not only decreased 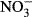-N leaching losses but also maintained cumulative maize yields.

The highest amount of yield-scaled nitrate leaching from the manure treatments could be attributed to high mineralization rate of organic N and mismatch between manure application and crop take-up in M treatments. These processes in the NCP are not clear, and further research is needed to assess factors influencing yield-scaled 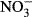-N leaching from manure application in wheat–maize doubling system and to improve fertilization management. On the other hand, although organic source as poultry manure can still have environmental impact and caution needs to be taken, straw returned with decreased mineral N fertilizer treatment (CTS and NTS) is a promising alternative with regard to yield-scaled 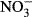-N leaching while increasing soil organic matter inputs in wheat–maize double-cropping system.

It is noteworthy that we discussed only the combined effects of tillage systems and N management regimes on crop yields and yield-scaled 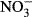-N leaching losses. Crop yield is one of the main financial components of farming. Farmers focus on the balance between costs and income. In recent years, fertilizers and land preparation have now become the higher direct cost in the winter wheat/maize double-cropping system in the NCP (Kang 2012). In spite of marginal reduction of wheat yield calculated as above, the decreased urea inputs (90 kg N ha^−1^ for winter wheat, 45 kg ha^−1^ for maize, respectively), together with the decreased costs of land preparation with no-tillage, make it feasible for farmers to implement the NTS and NTM treatments. Provided with significantly (P < 0.01) lower 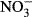-N losses than those of the CTU treatment over the 5 years, the NTS treatment also proved to be a potential alternative of the CTU treatment which is currently the most common management practices in the NCP. Economic analysis is needed in further study.

## Conclusions

This study demonstrated that the influence of tillage on crop yield depended on crop type under the semihumid temperate climate of the NCP. The effect of tillage on maize crop yields was insignificant throughout the 5-year rotation, while NT decreased wheat grain yield only for 2 of 5 seasons. However, similar crop yields between nitrogen management regimes on winter wheat and maize crop yields for the 5 rotation years suggest that it may be possible to decrease the rates of urea application while sustaining crop grain yields by adopting S and M regimes in place of the U regime in the area studied.

The tillage and N management regimes had significant effect on the yield-scaled 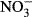-N leaching losses with CTS and NTS being lowest and NTM and CTM being highest in wheat and maize system, respectively. Thus, CTS minimized 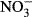-N leaching without losing production compared to CTU, and NTS may be a potential alternative to decrease yield-scaled 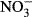-N leaching losses but not lower crop yield for the wheat–maize double-cropping systems in the NCP.
